# Supplementary material for: The undue influence of genetic information on senior medical students’ treatment decisions
Source: BMC Med Educ. 2023 Dec 8;23:938. doi: 10.1186/s12909-023-04895-w (PMC10709879; doi:10.1186/s12909-023-04895-w)
Supplement: Supplementary file 1 — Additional file 1: Appendix 1. [file 12909_2023_4895_MOESM1_ESM.docx]

**Appendix 1 – Phase 1 Questions**

**Stem 1**

Mary Sullivan is a 25 years-old female who presents to your GP surgery complaining of a 3-month worsening history of tiredness, intermittent diarrhoea, and generally “not feeling 100%”. Mary is worried because she has had the symptoms for a while now and asks, “could it be cancer?”. She has recently moved here from Adelaide, and hands you a summary of her medical history from her previous GP. You notice that she has previously had some genetic testing and has the HLADQ2 gene.

Please answer the following questions on gastroenterology, and Mrs Sullivan’s clinical presentation?

**Stem 2**

Mary Sullivan is a 25 years-old female who presents to your GP surgery complaining of a 3-month history of tiredness, intermittent diarrhoea, and generally “not feeling 100%”. Mary is worried because she has had the symptoms for a while now and asks, “could it be cancer?”. She has recently moved here from Adelaide, and hands you a summary of her medical history from her previous GP.

Please answer the following questions on gastroenterology, and Mrs Sullivan’s clinical presentation?

Q1 Which of the following is TRUE with regards to the enteric nervous system?

1. The neurotransmitter noradrenaline stimulates peristalsis
2. Substance P relaxes the circular muscle
3. The vagus nerve has parasympathetic nervous system activity from oesophagus to the descending colon
4. Enterochromaffin cells are basal granular cells which release 5-HT with mucosal stimuli
5. It is situated predominantly between the serosa and the longitudinal muscle layer

Q2 Regarding the normal composition of faeces, which of the following is TRUE?

1. 75% is water
2. 40% is solid material
3. 50% of the solid material is dead bacteria
4. 10% of the solid material is protein
5. 50% of the solid material is fat

Q3 Which of the following is correct with regards to lipid digestion and absorption?

1. Pancreatic lipase causes hydrolysis of triglycerides
2. Fat malabsorption is confirmed when faecal fat is >3% of ingested fats
3. Approximately 60% of bile salts that enter the gut are reabsorbed in the terminal ileum
4. Micelles present triglycerides to the luminal membrane for absorption
5. Fat digestion fails when pancreatic function falls to < 20%

Q4 Regarding absorption in the gastrointestinal tract, which statement is TRUE?

1. Ethanol is absorbed after reaching the small intestine
2. Iron is absorbed in the proximal bowel as Fe^3+^
3. Sodium and glucose co-transporter on enterocytes is cAMP dependent
4. Carbohydrate absorption occurs predominantly in the distal ileum and proximal colon
5. Vitamin B12 is absorbed in the duodenum

Q5 Regarding pancreatic exocrine functions, which of the following is FALSE:

1. Trypsin inhibitor protects the pancreas from self-digestion
2. Zymogens are stored and released from pancreatic acinar cells
3. Trypsinogen is activated by enterokinase
4. Carboxypeptidases are involved in carbohydrate and protein digestion
5. Pancreatic insufficiency develops when 98% of the exocrine pancreas is lost

Q6 Regarding gastrointestinal tract motility, which of the following is TRUE?

1. Conscious muscular control of motility occurs at mouth, upper oesophageal sphincter, striated muscles of proximal oesophagus and anus
2. There is longer lag time in gastric emptying for liquids than solids to allow better absorption
3. Colonic High Amplitude Propagated Contractions occur during sleep
4. Phasic contractions allow the proximal stomach to act as storage
5. The main motility function of the small bowel is storage

Q7 Regarding this patient’s presenting history and symptoms, the next appropriate investigation would be?

1. Stool culture
2. Anti-tTG IgA antibody
3. Small bowel biopsy
4. Full Blood Count (FBC)
5. CT abdomen

Q8 Small intestine histological structures include all the following, EXCEPT?

1. Microvilli on intestinal side of enterocytes
2. Villi with a core of vasculature and lymphatics
3. Intraepithelial lymphocytes – predominantly T cells
4. Paneth cells with a defensive function
5. Crypts lined with columnar epithelium

Q9 Regarding this patient’s presenting history and symptoms, the most appropriate management would be?

1. Rehydration and recovery
2. Antibiotics
3. Referral to a dietician
4. Gluten free diet
5. Loperamide

Q10 In a patient presenting with symptoms that might suggest irritable bowel syndrome, which of the following would be considered a “red flag” that warrants further investigation?

1. A mixture of both diarrhoea and constipation
2. Symptoms developing < 45 years of age
3. Urgency and or feeling of incomplete evacuation
4. Nocturnal diarrhoea
5. History of anxiety/depression
